# First Episode Psychosis and Schizophrenia Are Systemic Neuro-Immune Disorders Triggered by a Biotic Stimulus in Individuals with Reduced Immune Regulation and Neuroprotection

**DOI:** 10.3390/cells10112929

**Published:** 2021-10-28

**Authors:** Michael Maes, Kitiporn Plaimas, Apichat Suratanee, Cristiano Noto, Buranee Kanchanatawan

**Affiliations:** 1 Department of Psychiatry, Faculty of Medicine, Chulalongkorn University, Bangkok 10330, Thailand; drburanee@gmail.com; 2 Department of Psychiatry, Medical University of Plovdiv, 4000 Plovdiv, Bulgaria; 3 IMPACT Strategic Research Center, Deakin University, Geelong 3220, Australia; 4 Advanced Virtual and Intelligent Computing (AVIC) Center, Department of Mathematics and Computer Science, Faculty of Science, Chulalongkorn University, Bangkok 10330, Thailand; kplaimas@gmail.com; 5 Department of Mathematics, Faculty of Applied Science, King Mongkut’s University of Technology North Bangkok, Bangkok 10800, Thailand; apichat.s@sci.kmutnb.ac.th; 6 GAPi (Early Psychosis Group), Universidade Federal de São Paulo (UNIFESP), São Paulo 04021-001, Brazil; csnoto@gmail.com; 7 Schizophrenia Program (PROESQ), Department of Psychiatry, Universidade Federal de São Paulo (UNIFESP), São Paulo 04021-001, Brazil

**Keywords:** schizophrenia, neuro-immune, inflammation, physiological stress, bacterial translocation, psychiatry, LPS

## Abstract

There is evidence that schizophrenia is characterized by activation of the immune-inflammatory response (IRS) and compensatory immune-regulatory systems (CIRS) and lowered neuroprotection. Studies performed on antipsychotic-naïve first episode psychosis (AN-FEP) and schizophrenia (FES) patients are important as they may disclose the pathogenesis of FES. However, the protein–protein interaction (PPI) network of FEP/FES is not established. The aim of the current study was to delineate a) the characteristics of the PPI network of AN-FEP and its transition to FES; and b) the biological functions, pathways, and molecular patterns, which are over-represented in FEP/FES. Toward this end, we used PPI network, enrichment, and annotation analyses. FEP and FEP/FES are strongly associated with a response to a bacterium, alterations in Toll-Like Receptor-4 and nuclear factor-κB signaling, and the Janus kinases/signal transducer and activator of the transcription proteins pathway. Specific molecular complexes of the peripheral immune response are associated with microglial activation, neuroinflammation, and gliogenesis. FEP/FES is accompanied by lowered protection against inflammation, in part attributable to dysfunctional miRNA maturation, deficits in neurotrophin and Wnt/catenin signaling, and adherens junction organization. Multiple interactions between reduced brain derived neurotrophic factor, E-cadherin, and β-catenin and disrupted schizophrenia-1 (DISC1) expression increase the vulnerability to the neurotoxic effects of immune molecules, including cytokines and complement factors. In summary: FEP and FES are systemic neuro-immune disorders that are probably triggered by a bacterial stimulus which induces neuro-immune toxicity cascades that are overexpressed in people with reduced anti-inflammatory and miRNA protections, cell–cell junction organization, and neurotrophin and Wnt/catenin signaling.

## 1. Introduction

In 1995, Smith and Maes [1] launched the monocyte-T lymphocyte theory of schizophrenia, which incorporated neurodevelopmental factors and activation of immune pathways into a first comprehensive theory of schizophrenia. This theory considered that a neurodevelopmental pathology due to prenatal bacterial or viral infections increases the vulnerability to a later immune-inflammatory hit whereby products of activated macrophages and T lymphocytes, including cytokines and tryptophan catabolites (TRYCAT), result in neurotoxic effects on brain cells [1]. The presence of an inflammatory process in schizophrenia was first reported in 1997, when Maes et al. [2] showed increased plasma levels of complement component 3 (C3C) and C4, positive acute phase proteins, including haptoglobin (Hp) [2], and interleukin (IL)-6 [3], one of the cytokines which initiates the acute phase response in schizophrenia. These pioneering findings were replicated in later studies [4], which additionally showed immune-inflammatory processes in the CNS and associations between schizophrenia and immune genes, including single nucleotide polymorphisms in tumor necrosis factor (TNF)-α [5,6].

There is now evidence that schizophrenia phenotypes are accompanied by activation of the immune-inflammatory response system (IRS), including first episode psychosis (FEP), first episode schizophrenia (FES), multiple episode schizophrenia (MES), MES with worsening, the acute phase of schizophrenia, chronic schizophrenia, treatment resistant schizophrenia, deficit schizophrenia, and schizophrenia with comorbid mood symptoms and chronic fatigue-like symptoms [4,7]. In these different phenotypes, IRS activation with increased M1 macrophage, T helper (Th)-1, and Th-17 activation is accompanied by activation of the compensatory immune-regulatory system (CIRS), as indicated by increased levels of immune-regulatory pathways including Th-2 and T regulatory (Treg) cytokines, such as IL-10, IL-4, and IL-13, acute phase reactants, such as Hp, and TRYCAT levels [4,7]. As a consequence, the schizophrenia phenotypes present with a new homeostatic set point between both upregulated IRS and CIRS pathways, although there are indicants that the CIRS prevails in most phenotypes [4,7,8].

Studies on antipsychotic-naïve (AN)-FEP and FES patients are extremely important as the results may disclose the pathogenesis of the disease, and because the results are not affected by the effects of multiple episodes [8]. Moreover, the examination of AN-FEP may disclose causal pathways or molecular processes which are unaffected by the use of antipsychotics. AN-FEP is not only characterized by a cytokine storm with robust M1, Th-1, Th-17, Th-2, and Treg activation, but also by a greater IRS as compared with CIRS response [8]. Moreover, lowered CIRS protection due to relatively lower levels of the immune-regulatory soluble receptors (e.g., IL-2 receptor and TNF receptors) predicts a worse clinical outcome [8]. Furthermore, AN-FEP is accompanied by (a) lowered expression of brain-derived neurotrophic factor (BDNF), disrupted in schizophrenia 1 (DISC1), and ribonuclease III or double-stranded (ds) RNA-specific endoribonuclease (DROSHA) which exert neurotrophic, neuroprotective, and neurogenic functions and modulate microRNA (miRNA) biogenesis [9,10], and (b) lowered activity of paraoxonase (PON)-1, an enzyme with anti-inflammatory, anti-oxidant, and anti-microbial properties [11].

FES, which is the consequence of FEP, is characterized by (a) activated IRS/CIRS with increased levels of CCL11 (or eotaxin, a neurotoxic Th-2-associated cytokine), TRYCATs, oxidative stress indicants, and IL-10; (b) increased IgA C1q circulating immune complexes (CICs); (c) disorders in the expression of β-catenin, E-cadherin, plasmalemma vesicle associated protein (PLVAP), indicating dysfunctions in cell–cell, adherens, and vascular junctions; (d) increased IgA/IgM responses to Gram-negative bacteria, including Klebsiella pneumoniae [7]; and (e) lowered BDNF [12]. In FEP/FES, increased neurotoxicity due to M1, Th-1, and Th-2 activation contributes to a general cognitive decline (G-CoDe), which reflects abnormalities in brain connectome circuits [13].

As such, schizophrenia is an incredibly complex disease that involves the interaction of genetic factors, immune and intracellular signaling, metabolic pathways, and alterations in proteins/enzymes. Until now, the majority of genetic, pathway, and metabolic studies have concentrated on identifying and characterizing the individual genes, proteins, and metabolics involved in schizophrenia susceptibility. Nevertheless, genes/proteins with small to moderate effects interact to influence susceptibility and outcome in complex diseases such as schizophrenia. Network, pathway enrichment, and annotation analysis may provide insight into how genes and proteins interact to influence disease susceptibility [14]. Additionally, protein–protein interaction (PPI) network analysis may reveal novel drug targets for developing novel therapies that modulate pathways and transcription factors [14]. However, no study to date has delineated the characteristics of the PPI network of AN-FEP and its transition to FES, and the most significant paths, molecular patterns, cellular components, and diseases enriched in the PPI networks.

Hence, the aims of the current study were to delineate (a) the interactome or PPI network of FEP/FES and possible sub-networks; (b) the most influential core genes in the interactome, either hubs (disease causing genes) or bottlenecks (controlling the network); (c) the biological functions, pathways, molecular patterns, and cellular components that characterize FEP/FES; (d) the possible trigger factors of the FEP/FES interactome; and (e) the possible pathway similarities with other medical illness. 

## 2. Methods

### 2.1. Selection of Seed Proteins

This study is a secondary data analysis on existing data using open, deidentified, and non-coded datasets and, therefore, this is non-human subject research which is not subject to IRB approval. In our previous case-control studies conducted on Brazil and Thai patients with AN-FEP and FES, we identified significant biomarkers or differently expressed genes/proteins (DEPs) as compared with normal controls [7,9,10,15,16]. The patients were of both sexes and aged 18–65 years old. The socio-demographic and clinical data are displayed in [7,9,10,15,16]. In this study, we examined PPI networks of two related phenotypes, namely AN-FEP and the FEP/FES spectrum, with the latter combining AN-FEP and FES data. As such, this study provides information on the onset of schizophrenia (FEP) and the first episode, which is the direct sequel of FEP. Overall, 24 genes/proteins were selected in FEP/FES, namely (shown are the official gene symbols but not italicized): IL4; IL5; IL6; IL10; IL12A; IL13; IFNG (interferon-γ); TNF (tumor necrosis factor-α); CSF2 (Granulocyte-macrophage colony-stimulating factor); CCL3 (C-C motif chemokine 3); CCL11 (C-C motif chemokine 11 or eotaxin); IL1RN (IL-1 receptor antagonist); C1QA (Complement C1q); PON1 (paraoxonase1); CDH1 (cadherin-1); CTNNB1 (Catenin beta-1); PLVAP (Plasmalemma vesicle-associated protein); BDNF (brain-derived nuclear factor); COMT (Catechol O-methyltransferase); DROSHA (Ribonuclease III double-stranded (ds) RNA-specific endoribonuclease); DISC1 (disrupted in schizophrenia 1 protein); NDEL1 (Nuclear distribution protein nudE-like 1), and MBP (myelin basic protein). The downregulated proteins were: COMT, DISC1, DROSHA, CDH1, CTNNB1, PLVAP, BDNF, and PON1. All other genes were upregulated. There were fifteen FEP-associated seed genes, namely IL5, IL6, IL10, IL12A, IL13, IFNG, TNF, CSF2, NDEL1, MBP, PON1, BDNF, COMT, DROSHA, and DISC1 [7,8,9,10,11,16]. In the present study, we use different identification labels for the same molecules (e.g., IL-6 versus IL6) depending on whether we refer to genes (official PPI gene symbol, e.g., IL6) or proteins (IL-6). 

### 2.2. PPI Network Construction

Construction of the PPI network with network expansion was conducted using STRING version 11.0 (https://string-db.org, as accessed 19 September 2021). The STRING database was employed to assess the PPIs (minimum required interaction score was 0.400) and to construct zero-order (seed proteins only) and first-order (50 interactions in the first shell, none in the second shell) PPI networks among the genes (set organism is homo sapiens). We examined the network characteristics (number of nodes and edges, average number of neighbors, network diameter and radius, clustering coefficient, and network density, heterogeneity and centralization) of the zero and first-order networks as well as further expanded networks using STRING and Cytoscape (https://cytoscape.org, as accessed 19 September 2021) plugins including NetworkAnalyzer (Cytoscape App Store-NetworkAnalyzer, as accessed 19 September 2021). Hub nodes were authenticated as the top five nodes with the highest degree, and top non-hub bottlenecks as nodes with the highest betweenness centrality. Together they shape the backbone of the network.

Network clustering was carried out to cluster highly interconnected genes to identify protein commonalities with similar attributes and functions. In the present study, we employed STRING to perform Markov Clustering (MCL), which robustly identifies annotated complexes, and Cytoscape plugins, namely ClusterMaker. Molecular Complex Detection (MCODE) was performed using Metascape to detect smaller components of densely connected nodes which represent molecular complexes [17].

### 2.3. Enrichment Analysis

The list of seed and first-order genes (divided into communities by cluster analysis and up-and downregulated genes) were extended with known protein interactions from STRING, IntAct (https://www.ebi.ac.uk/intact/, as accessed 19 September 2021), GOnet (https://tools.dice-database.org/Gonet/, as accessed 19 September 2021), Metascape (http://metascape.org, as accessed 19 September 2021), inBio Discover (https://inbio-discover.com/, as accessed 19 September 2021), Enrichr (https://maayanlab.cloud/Enrichr/, as accessed 19 September 2021), or the R package ClusterProfiler 4.0 and examined for their pathway, function, or disease enrichment scores. In this study, we use the false discovery rate (FDR) corrected *p*-values. We searched the networks against GO biological processes (assemblies of molecular functions in pathways), GO molecular functions (protein activities at the molecular level), GO cellular component (location of the proteins) (www.geneontology.org, as accessed 19 September 2021), Kyoto Encyclopedia of Genes and Genomes (KEGG) pathways (https://genome.jp/kegg/, as accessed 19 September 2021), REACTOME (the European Bio-informatics Institute pathway database) (https://reactome.org, as accessed 19 September 2021), Translational Regulatory Relationships (TTRUST) (www.grnpedia.org, as accessed 19 September 2021), and DOID human disease phenotypes (Disease Ontology-Institute for Genome Sciences @ University of Maryland (disease-ontology.org, as accessed 19 September 2021). Moreover, Metascape was used to delineate and visualize the GO biological pathways and molecular processes, and PANTHER (PANTHER-Gene List Analysis (pantherdb.org), as accessed 19 September 2021), REACTOME, KEGG, and Wiki (WikiPathways-WikiPathways, as accessed 19 September 2021) pathways, which are over-represented in the gene sets. Metascape automatically clusters, hierarchically, the significant terms into a tree based on Kappa-statistical similarities among their gene memberships. This is useful because GO terms heavily overlap and, therefore, output GO terms show a large degree of redundancy. The top 10 performing terms obtained with Enrichr are displayed as bar graphs produced with Appyters.

### 2.4. Annotation Analysis and Annotation Visualization

Annotation analysis was employed to examine GO terms annotating the genes/proteins assembled into functional sets. GOnet analysis was employed to construct interactive graphs which, at the same time, contain genes and GO terms. Furthermore, we made custom GO term annotation lists consisting of the most representative GO terms (by preference, the significant children). inBio Discover was used to show the network with the top 4 selected DOID annotations. R package ClusterProfiler was employed to make dot plots of annotated GO term leaves, with size of the dots indicating gene number and color of the dots indicating the *p*-values.

## 3. Results

### 3.1. The PPI Network Topography of FEP and FES/FEP

**Figure 1:**
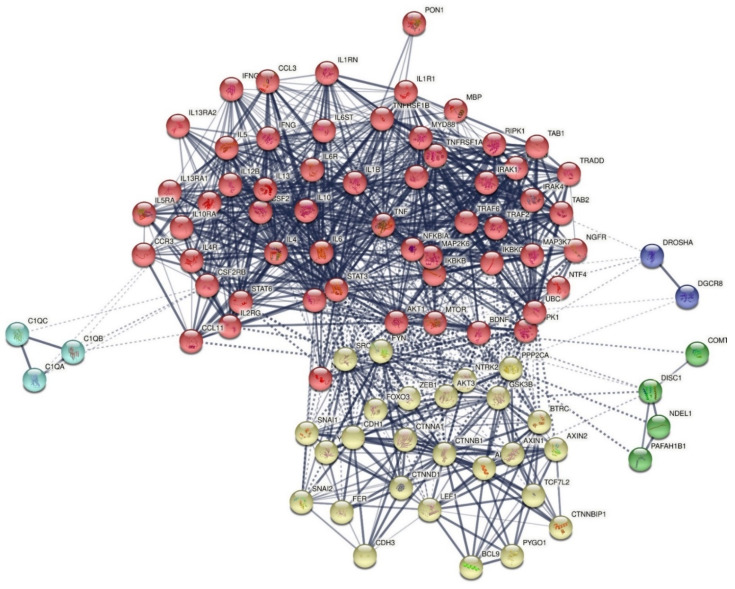
First order protein network showing 92 nodes and 1063 edges. MCL cluster analysis detected two clusters: (1) a first immune cluster (red color) was centered around BDNF, CCL11, CCL3, CSF2, IFNG, IL10, IL12A, IL13, IL1R1, IL4, IL5, IL6, MBP, PON1, and TNF, and (2) a second adhesion-associated cluster (yellow nodes) was centered around CDH1 and CTNNB1.

All genes/proteins were used to construct an undirected network representing the protein interactions of FEP/FES. The zero-order network consisted of 23 nodes, and the number of edges (*n* = 88) exceeded the expected number of edges (*n* = 24), with p-enrichment value of 1.0 × 10^−16^, and average node degree = 7.65 and average local clustering coefficient = 0.709. There were three singletons, namely PLVAP, DROSHA, and C1QA. Figure 1 displays the first order protein network that showed one singleton (PLVAP) and 92 nodes; the number of edges (*n* = 1063) exceeded the expected number of edges (*n* = 273) with p-enrichment value of 1.0 × 10^−16^, average node degree = 23.1, average local clustering coefficient = 0.678, average number of neighbors = 23.109, network diameter = 4 and radius = 2, characteristic path length = 1.919, network density = 0.254, and heterogeneity = 0.586.

Hub and betweenness analysis showed that IL6 (degree = 62), TNF (58), IL10 (46), IL4 (42), and CSF2 (39) were the top five hubs, while CTNNB1 (betweenness centrality = 0.0449), BDNF (0.0285) and CDH1 (0.014) were the top three non-hub bottlenecks. Top rank hubs and bottlenecks (computed as z degree + z betweenness) computed for the selected 23 seed proteins in a further enlarged network showed that the most influential proteins were in descending order of importance: CTNNB1, IL6, TNF, CDH1, IL4, IL10, and BDNF.

Figure 1 shows the results of MCL cluster analysis with an inflation parameter = 2.5. Two protein communalities were detected: (1) a first immune cluster was centered around CCL11, CCL3, CSF2, IFNG, IL10, IL12A, IL13, IL1R1, IL4, IL5, IL6, MBP, PON1, TNF, and BDNF; and (2) a second cell–cell junction-associated cluster was centered around CDH1 and CTNNB1. In the first-order network, there were two switches connecting these clusters. The first bridge was CDH1, which belongs to cluster 2 and is connected with CTNNB1 and with five seed genes in cluster 1 (CSF2, IL4, TNF, IL10, and IL6). In the first-order network, CHD1 shows 11 connections with cluster 1 seed genes and 16 with cluster 2 seed genes. The second switch was BDNF, which belongs to cluster 1 and shows interconnections with five cluster 1 genes, namely IL6 (0.811), TNF (0.805), IL4 (0.695), IL10 (0.613), and IFNG (0.419) and with one cluster 2 gene, namely CTNBB1 (0.932). In the first-order network, BDNF shows 16 connections (at >0.40) with cluster 1 genes and 7 with cluster 2 genes. More specifically, BDNF shows interconnections at >0.900 with four cluster 1 genes, namely STAT3 (0.967), TRAF6 (0.926), NTF4 (0.993), and NGFR (0.996), and three cluster 2 genes, namely NTRK2 (0.998), CTNNA1 (0.910), and CTNND1 (0.907). Moreover, BDNF showed interactions with COMT (0.733) and DISC1 (0.640) which do not belong to either cluster 1 or 2. We observed that AKT1, which belongs to cluster 1 in the first-order network, might be another switch, as it was interconnected with 11 cluster 1 seed proteins and with CTNNB1 and CDH1.

The seed proteins were used to construct a PPI network representing the protein interactions in FEP only. The first-order network (first shell) shows 65 nodes, the number of edges (*n* = 811) exceeded the expected number of edges (*n* = 193) with p-enrichment value of 1.0 × 10^−16^, with average node degree = 25, average local clustering coefficient = 0.75, average number of neighbors = 24.954, network diameter = 4 and radius = 2, characteristic path length = 1.780, network density = 0.390, and heterogeneity = 0.549. The top five hubs are, in descending order of importance, IL6 (87), TNF (85), IL10 (71), IL4 (71), and IFNG (68). BDNF was the most important bottleneck (0.0563), followed by IL6 (0.0473) and TNF (0.046).

### 3.2. Enrichment Analysis in FEP

**Figure 2:**
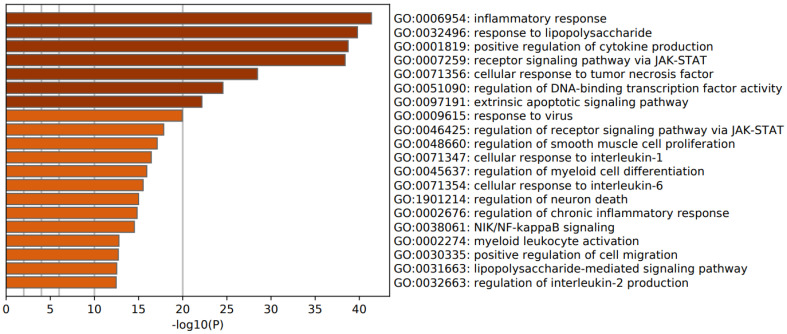
Heat map of enriched GO terms showing the top 20 biological functions overexpressed in the upregulated proteins of patients with first episode psychosis (accumulative hypergeometric *p*-values).

**Figure 3:**
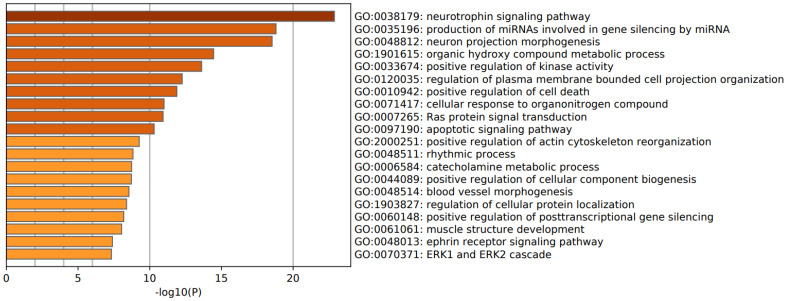
Heat map of enriched GO terms showing the top 20 biological functions associated with the downregulated proteins of patients with first episode psychosis (accumulative hypergeometric *p*-values).

Figure 2 (upregulated genes) and Figure 3 (downregulated genes) show heat maps (bar graphs) with the top 20 biological function GO enriched terms in FEP (shown are accumulative hypergeometric *p*-values). Figure 2 shows that the most important GO terms over-represented in the upregulated genes were: inflammatory response and cytokine production, and response to LPS and receptor signaling pathway via JAK-STAT. Figure 3 shows that the most important GO terms over-represented in the downregulated genes were: neurotrophin signaling pathway, production of miRNA involved in gene silencing, and neuron projection morphogenesis.

Table 1 shows the biological interpretation of the MCODE analysis performed using multiple databases (GO biological and molecular, KEGG, WikiPaths, PANTHER, and REACTOME gene sets) in FEP. MCODE performed on the upregulated genes in FEP detected one cluster which represented a response to LPS, response to molecule of bacterial origin, and inflammatory response. A second MCODE analysis conducted on the upregulated genes revealed two molecular complexes, namely signaling by interleukins and a second, which is shown in Table 1, representing TNFR1-induced NFκB signaling pathway, death receptor signaling, and TNF signaling. MCODE performed on the downregulated genes in FEP detected two small complexes, which represented: (1) neurotrophin/tropomyosin related kinase B (TrkB) signaling pathway and cellular component morphogenesis; and (2) RNA interference, production of miRNAs involved in gene silencing by miRNA, and production of small RNA involved in gene silencing by RNA. The search for a regulatory relationship using TTRUST (in Metascape) shows that the top two over-represented transcriptional networks in the upregulated FEP network are regulated by RELA (TRR0115, log10 *p* = −38) and NFKB1 (TRR00875, log10 *p* = −34).

### 3.3. Enrichment Analysis in FEP/FES

**Figure 4:**
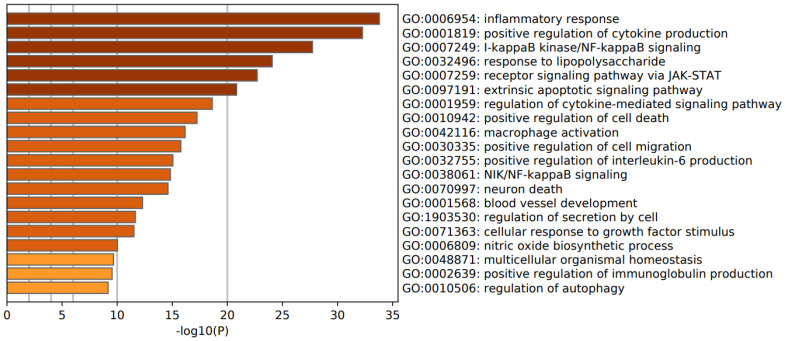
Heat map of enriched GO terms showing the top 20 biological functions in the first FEP/FES gene cluster (accumulative hypergeometric *p*-values).

**Figure 5:**
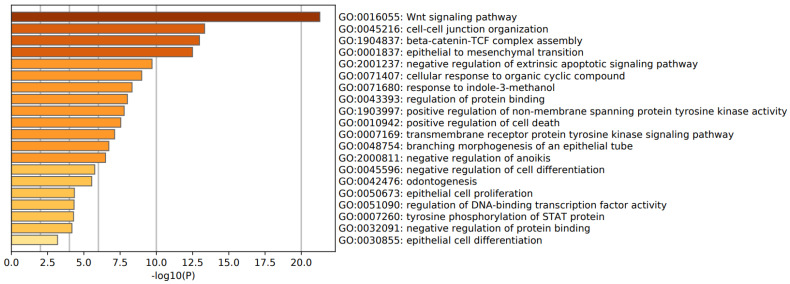
Heat map of enriched GO terms showing the top 20 biological functions in the second FEP/FES gene cluster (accumulative hypergeometric *p*-values).

Figure 4 shows a heat map with the top 20 biological GO enriched terms in the first FEP/FES cluster, indicating that the most important over-represented terms were: inflammatory response, positive regulation of cytokine production, I-kappa kinase/NFκB signaling, and a response to LPS. Figure 5 shows a heat map with the top 20 biological GO enriched terms in the second cluster of FEP/FES, namely Wnt signaling pathway, cell–cell junction organization, and beta-catenin-TCF complex assembly. Table 2 shows the top GP enriched terms in the complement factors, DISC1 and DROSHA.

Electronic Supplementary File (ESF) Figure S1 shows a bar graph with the top 10 performing cellular GO terms which were accumulated in FEP/FES genes, indicating that the catenin complex, and cytoplasmatic and vesicle membranes were the most significant cellular terms. ESF Figure S2 shows the top 10 performing REACTOME terms accumulated in the FEP/FES gene list, indicating that the first terms pointed towards an immune response and that the top 7–10 terms pointed towards different aspects of Toll-Like Receptor (TLR) 3/4 signaling. ESF Figure S3 displays the top 10 PANTHER terms, which were over-expressed in the FEP/FES gene list with CCKR, TLR, apoptosis, Wnt, and cadherin signaling as top pathways. ESF Figure S4 displays the top 10 WikiPathway terms which were over-expressed in the FEP/FES gene list, including TLR4 signaling and miRNA involvement. 

Table 1 shows the results of a MCODE analysis performed on all FEP/FES genes and detected a molecular complex (with core genes IFNG, IL6, CCL3, IL4, IL12A, and IL13), which represented microglial cell activation, positive regulation of tyrosine phosphorylation of STAT protein, and regulation of tyrosine phosphorylation of STAT protein. Annotation analysis also shows that a neuroinflammatory response (GO:0150076, pFDR = 1.0 × 10^−7.13^) was accumulated in the seed gene list selection. It is interesting to note that behavior (GO:0007610; log10 *p* = −4.941; 10 overlapping genes) and learning and memory (GO:0007611; log10 *p* = −4.908; 7 overlapping genes) accumulated in the FEP/FES genes.

ESF Figure S5 shows a network built with OmicsNet (using InAct; 2581 nodes, 4154 edges, 86 seeds). The top three over-represented transcriptional TTRUST networks were: SP1 (132 hits), NFKB1 (95), and RELA (95).

### 3.4. Annotation Analysis and Visualization

**Figure 6:**
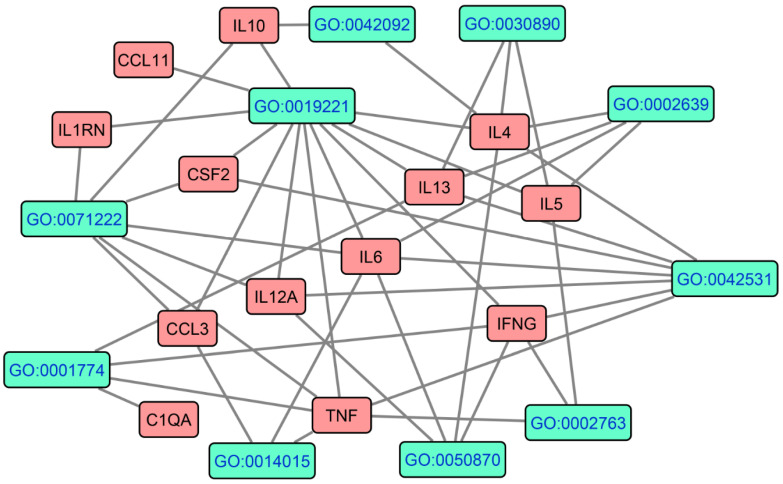
Results of GOnet annotation visualization in FEP/FES depicting the hierarchical structure of GO terms and the upregulated genes. This figure shows the seed protein nodes and the top 10 GO terms (i.e., their most important children).

Figure 6 shows the results of GOnet annotation visualization in FEP/FES, depicting the seed protein nodes and the top 10 GO terms (or their most important children). As expected, the GO terms comprised key immune processes, including cytokine mediated signaling pathway (GO:0019221, pFDR < 1 × 10^−10^), type-2 immune response (GO:0042092, pFDR = 6.42 × 10^−4^), positive regulation of T cell activation (GO: 0050870, pFDR = 6.384 × 10^−4^), positive regulation of B cell proliferation (GO:0030890, pFRD = 2.062 × 10^−4^), positive regulation of immunoglobulin production (GO:0002639, pFDR = 3.00 × 10^−6^), and positive regulation of myeloid leukocyte differentiation (GO:0002763, pFDR = 4.389 × 10^−4^). Moreover, other important GO terms were: cellular response to LPS (GO:0071222, pFDR = 1.520 × 10^−8^), microglial cell activation (GO:0001774, pFDR = 8.875 × 10^−7^), positive regulation of gliogenesis (pFDR = 7.962 × 10^−4^), and positive regulation of tyrosine phosphorylation of STAT protein (GO:0042531, pFDR = 1 × 10^−10^).

**Figure 7:**
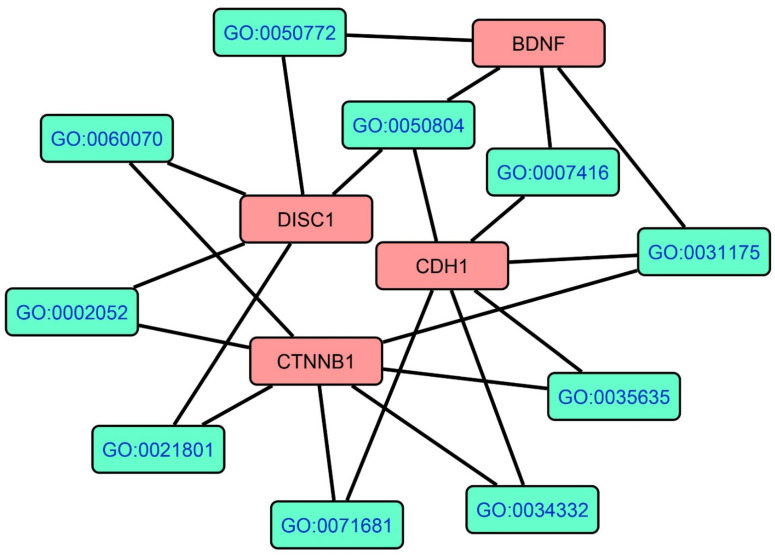
Results of GOnet annotation visualization in FEP/FES depicting the hierarchical structure of GO terms and the downregulated genes. This figure shows the seed protein nodes and the top 10 GO terms (i.e., their most important children).

Figure 7 shows the GOnet annotation analysis results with the GO terms and the four downregulated genes in FEP/FES. The GO term comprised canonical Wnt signaling pathway (GO:0060070, pFDR = 3.291 × 10^−2^), entry of bacterium into the host cell (GO:0035635, pFDR = 3.155 × 10^−3^), adherens junctions organization (GO:0034332, *p* = 3.291 × 10^−2^), modulation of chemical synaptic transmission (GO:0050804, pFDR = 1.990 × 10^−2^), synapse assembly (GO:0007416, pFDR = 3.497 × 10^−2^), neuron projection development (GO = 0031175, pFDR = 3.600 × 10^−2^), positive regulation of axonogenesis (GO:0050772, pFDR = 3.291 × 10^−2^), positive regulation of neuroblast proliferation (GO:0002052, pFDR = 9.751 × 10^−3^), cerebral cortex radial glia guided migration (GO:0021801, pFDR = 9.751 × 10^−3^), and cellular response to indole-3-methanol (GO:0071681, pFDR = 1.69 × 10^−3^).

**Figure 8:**
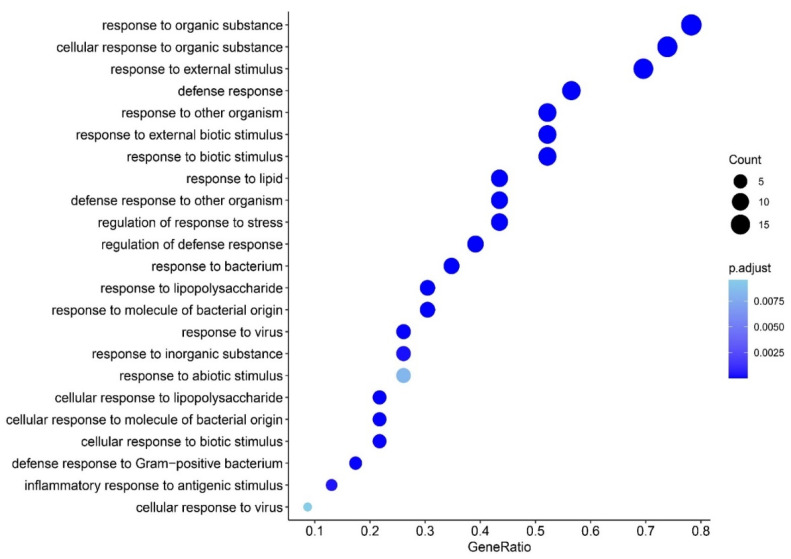
Results of annotation analysis using a custom-made GO list exploring the responses, cellular, or defense responses to a variety of mainly biotic stressors.

Figure 8 shows the results of annotation analysis using R package ClusterProfiler and a custom-made GO list comprising parent and child terms exploring response, cellular, or defense response to a variety of stressors, with the aim to differentiate the type of responses, including biotic responses. We observed that the most important over-represented GO terms in the FEP/FES gene list were responses to other organisms, external biotic stimulus, response to lipid, response to bacterium, and response to LPS, whereas there was much less or no evidence for a response to a virus or other biotic (fungal, parasites) or abiotic stimuli.

Consequently, we explored the network of interrelated genes using inBio Discover to delineate which diseases (DOID) are over-represented in the query gene list. Table 3 shows the top nine DOID disease annotations. This table indicates that the proteins involved in FES/FEP are enriched in a number of immune diseases, including immunodeficiency disease and autoimmune disease, and three gastro-intestinal diseases, namely intestinal disease, inflammatory bowel disease, and colitis. ESF Figure S6 shows the extended network and those four of the nine top annotations that were over-represented. The nodes of the extended PPI network involved in immune system and autoimmune disease and inflammatory bowel and intestinal disease are colored in red, orange, blue, and green, respectively. ESF Figure S7 shows all nodes (in red color) which were accumulated in intestinal disease (DOID:5295). ESF Figure S8 shows the results of an annotation analysis for all differentially expressed proteins, using R package ClusterProfiler and the GO list consisting of only leaf children in biological processes.

## 4. Discussion

### 4.1. The PPI Network of FEP/FES

The first major finding of this study is that we were able to construct zero and first-order PPI networks of FES/FEP which show high connectivity and some unexpected interactions, including the central role of BDNF. We found that the backbone of the FEP network is shaped by six genes (IL6, TNF, IL10, IL4, IFNG, and BDNF) and the backbone of FEP/FES by IL6, TNF, IL10, CCTNB1, CDH1, and BDNF. Moreover, our network analysis revealed two major protein communalities, namely one centered around immune-inflammatory genes and BDNF, and a second cluster around cell–cell junction genes (CTNNB1 and CDH1). Most importantly, BDNF appears to function as a switch between both communalities, as it shows interconnections with immune genes and CTNNB1 and the high-affinity receptor of BDNF, namely neurotrophic tyrosine kinase receptor 2 (NTRK2), which was allocated to the cell–cell junction cluster. Another, smaller, bridge between both communalities is CDH1, which is interconnected with CTNNB1 and with CSF2, IL4, TNF, IL10, and IL6.

Therefore, it appears that an immune response, as observed in FEP/FES, comprises not only IRS and CIRS components [4], but also different neurotrophic (BDNF, NTF4, and NGFR) and cell adhesion (CTNNB1 and CDH1) factors. By inference, the neurotoxic effects of the immune response may be counterbalanced by CIRS (Th-2 and Treg) [7], neuroprotective, and cell–cell adhesion genes. It is tempting to speculate that the participation of neuroprotective and cell–cell adhesion genes in this immune network is another conserved regulatory process protecting against detrimental neuro-immune effects.

### 4.2. Trigger Factors in FEP and FEP/FES

GO annotation and MCODE analysis revealed that the upregulated genes in FEP and the cluster-1 genes in FEP/FES were highly significantly associated with a response to a molecule of bacterial origin and a response to LPS. In fact, a response to LPS was the second most important GO term overexpressed in the upregulated proteins in FEP. Moreover, annotation analysis using a custom-made GO list with possible “responses to…” or “cellular responses to …” or a “defense response to…” showed that the most significant paths enriched in the network were responses to an organic substance (determined as cytokine or lipid), an external stimulus, response to other organisms, or an external biotic stimulus. Further analysis showed that a response to a bacterium and LPS were the most significant paths enriched in the FEP/FES network, whereas a response to a virus was less significant and did not appear in the GO and MCODE enrichment analysis. Moreover, an abiotic stimulus and fungal or parasite stimuli were not significantly enriched.

All in all, it appears that FEP and FEP/FES may be triggered or maintained by a response to LPS of Gram-negative bacteria. These findings corroborate a recent study which reported increased IgA/IgM responses to LPS of Gram-negative bacteria in FES and deficit schizophrenia, and a significant association between these indicants of bacterial load and the G-CoDe (general cognitive impairments) and symptom profiles, especially psychosis [7,18]. Such findings may indicate increased bacterial translocation in FES/FEP through leaky gut (increased gut permeability) with deficits in tight and adherens junctions and the vascular barrier, as well [7]. Interestingly, three of the top nine DOID annotations which were overrepresented in the PPI network of FEP/FES comprised intestinal annotations, including intestinal disease, inflammatory bowel disease, and colitis.

### 4.3. Upregulated Pathways, Molecular and Cellular Processes in FEP

The third major finding of this study is that the upregulated genes in FEP were enriched in key pathways and cellular functions that play a key role in immune-inflammatory signaling, namely the receptor signaling pathway via Janus kinases/signal transducer and activator of transcription proteins (JAK-STAT) pathway, the TNFR1-induced NFκB signaling pathway, and TNF- and death receptor signaling. Moreover, STAT3 and STAT6 were prominent nodes in the first order network, with STAT3 occupying a central position. Many cytokines (e.g., IL-2, IL-4, IL-6, IL-10, IL-12, IFN-γ) signal via the JAK-STAT pathway, thereby transactivating Janus kinases leading to translocation of STATs to the nucleus and upregulation of cytokine-modifiable genes [19]. The JAK-STAT pathway plays a key role in inflammation, cell death, cell division, and polarization of T cells; STAT3 is associated with autoimmune responses, and STAT6 plays a key role in M2 macrophage activation and Th-2 differentiation with production of IL-4, IL-5, IL-9, and IL-13 [19,20,21]. Recently, Sharma et al. [22] reported that a subgroup of schizophrenia patients showed increased STAT1 levels.

Our enrichment analyses show that TNF plays an important role in FEP and suggests that TNF-induced IκB kinase (IKK) activation with consequent NFκB translocation to the nucleus is one of the key processes in FEP. Moreover, we found that both NFκB1 and RELA (NFκB p65 unit or transcription factor p65) are the most prominent transcription factors in the FEP network. RELA plays a role in NFκB activation and translocation of the released NFκB complex to the nucleoplasm and contributes to DNA binding in the NFκB complex [23]. This NFκB heterodimeric RELA-NFKB1 complex functions as a transcriptional activator and plays a key role in gene expression of multiple cytokines [23]. In schizophrenia, it was shown that increased NFκB activity may contribute to cortical immune activation [24]. In a Japanese population, schizophrenia is associated with variants of the *RELA* gene and has a significant effect on pre-pulse inhibition [25]. Importantly, this pathway is not only induced by TNF, but also by other cytokines and LPS [26]. Previously, we have reviewed the many neurotoxic effects of increased TNF-α levels in schizophrenia, and especially in deficit schizophrenia [27]. Moreover, the frequency of the *TNF2(A)* allele, which affects plasma TNF levels, is significantly increased in schizophrenia and *TNF2* homozygotes are detected in schizophrenia only [6].

### 4.4. Downregulated Pathways, Molecular and Cellular Processes in FEP

The fourth major finding is that the downregulated genes were enriched in the receptor protein tyrosine kinase (RTK) and neurotrophin/Trk receptor signaling pathways, cellular component morphogenesis, and production of miRNAs involved in gene silencing. RTK is a family of high-affinity cell surface receptors, including Trk, which is activated by neurotrophins such as BDNF [28]. Interestingly, RTKs regulate the threshold for macrophage activation, thereby promoting homeostasis and protecting tissues from inflammatory damage [29]. BDNF/Trk signaling contributes to axonal growth, axonal guidance, plasticity, dendritic arborization, synapse structure and formation and connections, neurogenesis, differentiation of new neurons and synapses, and axonal and dendritic sprouting [30]. Moreover, BDNF may have anti-inflammatory and anti-apoptopic effects via modulation of MyD88/NFκB and PI3K/AKT-signaling pathways [31]. Following bacterial infection, BDNF pretreatment reduces the expression of TNF-α, IL-16, IL-1β, and the NFκB pathways, and increases IL-10 and Trk expression [31]. LPS-associated inflammation alters BDNF/TrkB signaling in the hippocampus, nucleus accumbens, and prefrontal cortex in association with the onset of depressive behaviors [32]. A recent meta-analysis showed that schizophrenia is associated with reduced BDNF with a medium effect size (Hedges g = −0.458, *p* < 0.004), and that these effects are not influenced by the drug state of the patients [33]. Moreover, the interaction between *BDNF* and *NTRK2* gene polymorphisms may increase susceptibility to paranoid schizophrenia [34].

We found that the downregulated DISC1 gene is enriched in various GO biological terms indicating neurogenesis, axonogenesis, and axon extension. DISC1 is now established as a risk factor for schizophrenia and other major psychiatric illness and is involved in aspects of adult progenitor proliferation, neurogenesis, neurite outgrowth, cytoskeletal modulation, signal transduction, and CTNNB1 abundance [35,36]. It is interesting to note that inflammatory signaling via TLR3 is accompanied by impairments in dendritic spine and growth via effects on MYD88 and, consequently, DISC1 expression [37]. 

Another highly significant GO path enriched in the downregulated network was the “production of miRNAs involved in gene silencing by miRNA”, which may, at least in part, be ascribed to downregulation of DROSHA, which is a ribonuclease (RNase) III family enzyme and plays a key role in miRNA maturation [38]. In mammals, the miRNA network comprises 5000–10,000 miRNAs which regulate the expression of 60% of the protein-coding genes through translational silencing and mRNA destabilization [39,40]. Importantly, miRNA regulate the adaptive and innate immune response and act as fine-tuning regulators, preventing an overzealous inflammatory response and thereby maintaining homeostasis [40]. Many of the miRNAs which are associated with schizophrenia phenotypes [41,42] display immune regulatory effects. For example, miR-9 exerts a negative feedback on NFκB and is dysregulated in neural progenitor cells of schizophrenia patients [43]; miR-132 inhibits inflammation signaling (via acetylcholine, STAT3, and NFKB) and is dysregulated in schizophrenia [44]; miR-146 inhibits inflammatory responses and is downregulated in monocytes of postpartum psychosis patients [45]; and miR-149 inhibits LPS-induced inflammation (via STAT3, NFκB, TNF, IL-6) and is a candidate biomarker of psychiatric disease including bipolar disorders [46].

### 4.5. Pathways, Molecular and Cellular Processes in FEP/FES

The enrichment and annotation analysis revealed other important drug targets in FEP/FES. Firstly, we found that a neuroinflammatory response was enriched in the seed gene FEP/FES list, whilst MCODE showed that a cytokine/chemokine complex of IFN-γ, IL-6, IL-12A, CCL3, IL-4, and IL-13 was strongly associated with microglial cell activation and tyrosine phosphorylation of STAT proteins. These results extend the findings that schizophrenia is accompanied by microglial activation [47]. Moreover, the upregulated genes in FEP/FES were enriched in “the positive regulation of gliogenesis”. In adulthood, gliogenesis is maintained to renew oligodendrocytes; however, following inflammatory disease and injuries, gliogenesis becomes more active (reactive astroytosis or astrogliosis) and may have negative consequences, thereby contributing to immune-inflammatory responses and altering the balance between neurogenesis and gliogenesis [48].

Secondly, WikiPathway and PANTHER enrichment analysis revealed that the upregulated genes were strongly associated with the TLR signaling (especially TLR4) and tolerance pathways. These findings extend those of previous publications indicating activation of the TLR4 proinflammatory pathway in schizophrenia [49].

Third, GO annotation analysis revealed that the cluster 2 genes are enriched in the Wnt/catenin pathway and cell–cell junction organization. Moreover, different combinations of the downregulated genes were associated with the Wnt/catenin pathway (DISC1 and CTNNB1), adherens junctions organization (CDH1 and CTNNB1), synapse assembly (CDH1 and BDNF), neuron projection development (BDNF, CTNNB1 and CDH1), neuroblast proliferation (DISC1 and CTNNB1), cerebral cortex radial glia guided migration (DISC1 and CTNNB1), positive regulation of axonogenesis (BDNF and DISC1), and modulation of chemical synaptic transmission (BDNF, CDH1 and DISC1). 

CTNNB1 is a component of the Wnt/β-catenin signaling pathway and the E-cadherin-catenin adhesion complex, which play a key role in epithelial integrity and tissue architecture maintenance [50,51]. The Wnt/catenin pathway is strongly involved in neurogenesis, axonal spreading and branching, connectivity between pre- and post-synaptic neuronal regions, regulation of synaptic functions and modeling of synaptic structures, modulation of excitatory synaptic transmission, LTP, and post-synaptic protein assembly [52]. The Wnt/β-catenin signaling pathway also regulates immune-inflammatory responses and T-cell-inflammation [53,54,55]. Inflammatory responses, due to infections with pathogenic bacteria, may affect the Wnt/β-catenin signaling pathway [55] and the E-cadherin-catenin adhesion complex [56]. For example, in inflammatory bowel disease, impairments in the latter complex are affected by the inflammatory milieu and may cause dysregulations of the actin cytoskeleton leading to aberrations in intracellular signaling and transcriptional regulation [57]. Moreover, the Wnt/catenin pathway may regulate BDNF expression while these two pathways may have common effector actions [58], and *BDNF* polymorphisms are associated with changes in the Wnt/β-catenin pathway [59]. In hippocampal neurons, BDNF-disruption of cadherin-β-catenin complexes is associated with increased synapse density [60].

Disorders in the Wnt/catenin pathway were previously described in FES and schizophrenia [7,61,62] and alterations in E-cadherin and beta-catenin levels in FES are strongly associated with increased bacterial translocation [7]. This more generalized disorder in paracellular and cell-cell junctions in FEP/FES may, at least in part, be related with the increased frequency of the *Hp2* allele and the *Hp2.2* genotype (prehaptoglobin-2 or zonulin) [63] and increased zonulin levels [64]. Moreover, mutations in the *CTNNB1* gene (*c.1943 A>G*) are associated with schizophrenia [65], whilst *CTNNB1* KO mice display anxiety behaviors and *CTNNB1* KO in paraventricular interneurons accompanied by impairments in social interactions, repetitive behaviors, and object recognition [66]. Association, candidate gene, and genome-wide association studies show that cadherins may be involved in the pathophysiology of schizophrenia [67,68].

Fourth, exploration of transcriptional regulation showed that SP1, NFκB1, and RELA were the most prominent transcription factors in the FEP/FES network. SP1 or specificity protein 1 (or transcription factor Sp1) is a ubiquitously expressed transcription factor, which regulates the expression of a variety of house-keeping and tissue-restricted genes frequently involved in immune responses, response to DNA damage, and apoptosis [69]. This explains that SP1 is associated with the pathophysiology of some neurodegenerative and neuroinflammatory disorders, including Alzheimer’s and Huntington’s disease and multiple sclerosis [69]. Interestingly, neurons have a decreased capacity of activating NFκB, but κB cis elements may bind to SP1 [70] and SP1 interacts with RELA to form a complex [71]. In cortical neurons, SP1 is an oxidatively-induced transcription factor which regulates neuronal survival [72]. In Huntington’s disease, pathogenic SP1 cascades cause repression of neuronal genes [73]. Interestingly, both NFκB and SP1 modulate antimicrobial activity against Gram-negative bacteria [74].

Fifth, biological GO term classifications showed that complement factors were enriched not only in microglial activation and humoral immune responses, but also in synapse pruning and organization. The involvement of complement in FEP/FES agrees with previous findings showing increased plasma C3C and C4 and CSF and brain C4 complement factors in schizophrenia [2,75,76]. Moreover, increased C1qA, C3, and C4 transcripts were reported to be associated with microglial activation in the midbrain of schizophrenia patients [77]. It should be added that FES is accompanied by increased formation of the IgA C1qA CIC, which may have detrimental effects in its own right [7]. Nevertheless, the complement pathways established in FEP/FES are not a key component of the interactome and, therefore, should be considered as secondary phenomena. In addition, also PON1 was not a key component of the interactome and showed only a few interactions. Other CIRS components could not be included, such as natural IgM responses to oxidatively specific epitopes [7]. Both PON1 and natural IgM are first line innate immune defenses against bacterial infections and display strong anti-inflammatory and antioxidant properties, explaining that impairments in both systems contribute to FEP/FES [7].

### 4.6. Conclusions

**Figure 9:**
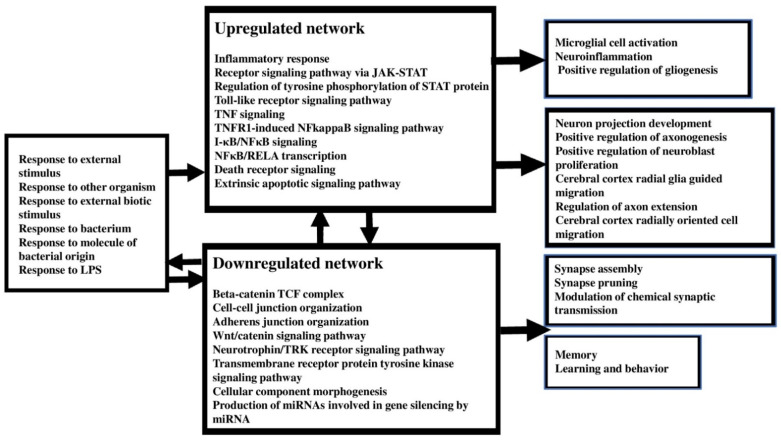
Summary of the findings of the enrichment/annotation analysis using upregulated and downregulated genes in first episode psychosis and schizophrenia.

Figure 9 shows a summary of the enrichment and annotation analysis reported in our study. All in all, the analyses suggest that increased bacterial translocation, in particular that of Gram-negative bacteria, is the most probable trigger and maintaining factor of FEP and FEP/FES, causing an immune-inflammatory milieu with involvement of TNF, NFκB/RELA, SP1, and JAK-STAT signaling, including tyrosine phosphorylation of STAT proteins, and death receptor and TLR4 signaling. A specific cytokine/chemokine complex consisting of IFN-γ, IL-6, IL-12A, CCL3, IL-4, and IL-13 is strongly associated with microglial activation and neuroinflammation, and this upregulated immune network additionally positively regulates gliogenesis. FEP/FES is also accompanied by lowered immune regulation and thus protection against an overzealous inflammatory process due to dysfunctional miRNA maturation, and deficits in neurotrophin/Trk, RTK and Wnt/catenin signaling.

By inference, individuals with reduced CIRS [4], including miRNA maturation and deficits in neurotrophin/Trk, RTK, and Wnt/catenin signaling, neuroprotection (including neurotrophin/Trk and Wnt/catenin signaling, lowered DISC1 expression, and interactions between reduced BDNF, CDH1, CTNNB, and DISC1 may be at an increased risk to develop FEP and FES as a consequence of an immune response following bacterial infections and the ensuing neuro-immune toxicity. The latter may cause a multitude of neuronal dysfunctions and cognitive impairments and schizophrenia behaviors [7], as shown in Figure 9.

Interestingly, almost all pathways or molecular patterns enriched in the interactome of FEP/FES are directly (NFκB/RELA, TLRs, TNF, adherens junctions and cell–cell junctions, complement activation with C1QA CIC formation, neurotrophin/Trk pathway, Wnt signaling, microglial activation, gliogenesis, neurotoxicity) or indirectly (JAK-STAT, DISC1, SP1) affected by LPS. As such, the increased LPS levels in FES may maintain peripheral and central immune activation resulting in neurotoxic effects on central neuronal circuitry, neurogenesis, and synapse functions [7]. Disorders in cell–cell junction organization may contribute to the maintenance of increased bacterial translocation and activation of complement cascades with increased IgA C1QA CIC formation, which may further fuel the immune response [7]. It should be added that LPS-induced maternal immune activation may have increased the vulnerability to such immune hits by inducing neurodevelopmental disorders with sensitized immune-inflammatory pathways (including in TLR4 and inflammasome) and lowered neuroprotection [78,79].

Future research in FEP should examine the microbiome, gut dysbiosis, and consequent bacterial translocation due to leaky gut as well as alterations in the microbiota–gut–brain axis [18], which may ultimately activate or dysregulate the many pathways discussed in the current review. In addition, it would be most informative to examine differences in the gut–brain axis between FEP, deficit schizophrenia, and other major psychiatric and psychosomatic disorders. There is some evidence that increased leaky gut and bacterial translocation and their impact on neuro-immune pathways may be a transdiagnostic phenomenon [80] which is detected in affective disorders [81] and Myalgic Encephalomyelitis [82]. Future research should examine the effects of new or repurposing drugs and natural anti-inflammatory and antioxidant compounds on the gut–brain axis and intracellular pathways in FEP (see Figure 9). For example, one could aim to attenuate the primary bacterial translocation in FEP by using minocycline, which has, additionally, anti-inflammatory and antioxidant capacities [83]. One could also aim to target NFκB using specific kinase inhibitors with or without natural NFκB inhibitors, such as curcuma [84]. However, a novel approach to treating FEP and, thus, preventing schizophrenia, its worsening course, and the onset of deficit syndrome appears to require a highly complex approach, as deficiencies in the CIRS and neurotrophic factors appear to be critical. As such, combination therapies with immunotherapies (including intravenous immunoglobulins, which have some efficacy in schizophrenia in case reports [85]) and neurotrophic factors [84] appear to be warranted.

## Figures and Tables

**Table 1:** Results of Molecular Complex Detection (MCODE) analysis performed on upregulated and downregulated differentially expressed proteins in first episode psychosis (FEP) and/or first episode schizophrenia (FES).

MCODE Components	GO ID	Biological Term	Log10 (*p*)
Upregulated genes in FEP, MCODE1 (Biological GO terms)	GO:0032496	response to lipopolysaccharide	−41.6
	GO:0006954	inflammatory response	−40.8
	GO:0002237	response to molecule of bacterial origin	−40.8
Upregulated genes in FEP, MCODE2 (GO, PANTHER, KEGG, WikiPaths)	R-HSA-5357956	TNFR1-induced NFkappaB signaling pathway	−10.1
	R-HSA-73887	death receptor signaling	−9.8
	R-HSA-75893	TNF signaling	−9.4
Downregulated genes in FEP, MCODE1 (Biological GO terms)	GO:0048011	neurotrophin TRK receptor signaling pathway	−22.5
	GO:0007169	transmembrane receptor protein tyrosine kinase signaling pathway	−22.1
	GO:0032989	cellular component morphogenesis	−21.8
Downregulated genes in FEP, MCODE2 (Biological GO terms)	GO:0016246	RNA interference	−15.6
	GO:0035196	production of miRNAs involved in gene silencing by miRNA	−15.5
	GO:0070918	production of small RNA involved in gene silencing by RNA	−15.3
All seed genes in FEP/FES, MCODE1 (Biological GO terms)	GO:0001774	microglial cell activation	−13.2
	GO:0042531	positive regulation of tyrosine phosphorylation of STAT protein	−12.3
	GO:0042509	regulation of tyrosine phosphorylation of STAT protein	−11.8

**Table 2:** Biological GO term classifications of differently expressed proteins in first episode psychosis or schizophrenia.

Top Genes	GO Term ID	GO Term Definition	*P*	*p* FDR Adjusted	Number of Genes
**Complement factors**					
1	GO:0098883	synapse pruning	1.00 × 10^−10^	1.41 × 10^−6^	3
2	GO:0030449	regulation of complement activation	2.02 × 10^−10^	1.11 × 10^−3^	3
3	GO:0002920	regulation of humoral immune response	3.18 × 10^−7^	1.17 × 10^−3^	3
4	GO:0006958	complement activation, classical pathway	5.40 × 10^−7^	1.30 × 10^−3^	3
5	GO:0002455	humoral immune response mediated by circulating immunoglobulin	5.92 × 10^−7^	1.30 × 10^−3^	3
6	GO:0006956	complement activation	7.29 × 10^−7^	1.34 × 10^−3^	3
7	GO:0016064	immunoglobulin mediated immune response	1.08 × 10^−6^	1.56 × 10^−3^	3
8	GO:0019724	B cell mediated immunity	1.13 × 10^−6^	1.56 × 10^−3^	3
9	GO:0002449	lymphocyte mediated immunity	2.35 × 10^−6^	2.66 × 10^−3^	3
10	GO:0050808	synapse organization	2.46 × 10^−6^	2.66 × 10^−3^	3
**DISC1**					
1	GO:0021799	cerebral cortex radially oriented cell migration	1.13 × 10^−8^	1.25 × 10^−4^	3
2	GO:0021795	cerebral cortex cell migration	3.82 × 10^−8^	1.40 × 10^−4^	3
3	GO:0045773	positive regulation of axon extension	3.82 × 10^−8^	1.40 × 10^−4^	3
4	GO:0022029	telencephalon cell migration	9.56 × 10^−8^	2.46 × 10^−4^	3
5	GO:0021885	forebrain cell migration	1.11 × 10^−7^	2.46 × 10^−4^	3
6	GO:0021954	central nervous system neuron development	2.54 × 10^−7^	4.67 × 10^−4^	3
7	GO:0050772	positive regulation of axonogenesis	3.06 × 10^−7^	4.81 × 10^−4^	3
8	GO:0030516	regulation of axon extension	4.42 × 10^−7^	6.09 × 10^−4^	3
9	GO:0061387	regulation of extent of cell growth	6.67 × 10^−7^	8.17 × 10^−4^	3
10	GO:0021987	cerebral cortex development	7.83 × 10^−7^	8.48 × 10^−4^	3
**DROSCHA**					
1	GO:0031053	primary miRNA processing	1.84 × 10^−7^	2.03 × 10^−3^	2
2	GO:0010586	miRNA metabolic process	1.18 × 10^−6^	5.95 × 10^−3^	2
3	GO:0035196	production of miRNAs involved in gene silencing by miRNA	2.08 × 10^−6^	5.95 × 10^−3^	2
4	GO:0070918	production of small RNA involved in gene silencing by RNA	2.70 × 10^−6^	5.95 × 10^−3^	2
5	GO:0031050	dsRNA processing	2.70 × 10^−6^	5.95 × 10^−3^	2

**Table 3:** Results of inBio Discover annotation analysis with the DOID disease annotations classification in first episode psychosis and schizophrenia.

DOID ID	Disease	Size	Overlap	Enrichment	*p*-Value
DOID:2914	immune system disease	1.9 k	89/246	3.81	2.4 × 10^−30^
DOID:612	primary immunodeficiency syndrome	1.3 k	70/246	4.28	2.9 × 10^−26^
DOID:5295	intestinal disease	1.0 k	61/246	4.89	9.2 × 10^−26^
DOID:0060032	autoimmune disease of the musculoskeletal system	645	48/246	6.05	4.2x 10^−24^
DOID:0050589	inflammatory bowel disease	306	35/246	9.30	8.7 × 10^−24^
DOID:417	autoimmune disease	1.1 k	58/246	4.42	3.0 × 10^−22^
DOID:9500	leukocyte disease	417	38/246	7.41	3.3 × 10^−22^
DOID:65	connective tissue disease	1.8 k	76/246	3.35	5.3 × 10^−22^
DOID:0060180	Colitis	237	30/246	10.29	8.8 × 10^−22^

## Data Availability

Data are openly available to the public in [7,8,9,10,11,16].

## Supplementary Materials

The following are available online at https://www.mdpi.com/article/10.3390/cells10112929/s1, Figure S1: Heatmap (top-10) of enriched GO cellular component terms accumulated in the differently expressed proteins in first episode psychosis and schizophrenia., Figure S2: Heatmap (top-10) of enriched Reactome terms over-represented in the differently expressed proteins in first episode psychosis and schizophrenia, Figure S3: Heatmap (top-10) of enriched Panther Pathways that were over-represented in the differently expressed proteins in first episode psychosis and schizophrenia. Figure S4: Heatmap (top-10) of enriched WikiPathways that were over-represented in the differently expressed proteins in first episode psychosis and schizophrenia. Figure S5: Protein-protein interaction network (built with OmicsNet and IntAct) of first episode psychosis and schizophrenia. The top-3 transcriptional factors are shown as blue spots (SP1, NFκB1, and RELA). Proteins in green and bundles in pink. Figure S6. Protein-protein interaction network (built with inBio Discover) of differently expressed proteins in first episode psychosis and schizophrenia. Shown are 4 top annotations that are over-represented in the gene list, namely: red color: immune system disease (DOID:2914); blue color: inflammatory bowel disease (DOID:0050589); green color: intestinal disease (DOID:5295); yellow color: autoimmune disease (DOID:417) Figure S7: Differently expressed proteins in the extended protein-protein interaction network that accumulated in intestinal disease (DOID:5295). Figure S8. GO functional enrichment analysis of all differently expressed proteins in first episode psychosis and schizophrenia using R package ClusterProfiler. Only annotated GO term leaves are shown in this figure. The x-axis shows the gene ratio and the y-axis the annotated GO biological terms. The size of the dots is proportional to the gene number; *p*-values of all GO terms are colored as indicated in the figure.
