# Supplementary material for: First Episode Psychosis and Schizophrenia Are Systemic Neuro-Immune Disorders Triggered by a Biotic Stimulus in Individuals with Reduced Immune Regulation and Neuroprotection
Source: Cells. 2021 Oct 28;10(11):2929. doi: 10.3390/cells10112929 (PMC8616258; doi:10.3390/cells10112929)
Supplement: Supplementary file 1 [file cells-10-02929-s001.zip › cells-1416449 supplementary after proof.pdf]

First episode psychosis and schizophrenia are systemic neuro-immune disorders triggered by a biotic stimulus in individuals with reduced immune regulation and neuroprotection.

Michael Maes, M.D., Ph.D. [a,b,c], Kitiporn Plaimas, Dr.rer.nat. [d], Apichat Suratanee, Dr.rer.nat. [e], Cristiano Noto, M.D. [f,g], Buranee Kanchanatawan, M.D. [a]

[a] Department of Psychiatry, Faculty of Medicine, Chulalongkorn University, Bangkok, Thailand

[b] Department of Psychiatry, Medical University of Plovdiv, Plovdiv, Bulgaria

[c] IMPACT Strategic Research Center, Deakin University, Geelong, Australia

[d] Advanced Virtual and Intelligent Computing (AVIC) Center, Department of Mathematics and Computer Science, Faculty of Science, Chulalongkorn University, Bangkok, 10330, Thailand

[e] Department of Mathematics, Faculty of Applied Science, King Mongkut's University of Technology North Bangkok, Bangkok 10800, Thailand

[f] GAPi (Early Psychosis Group), Universidade Federal de São Paulo (UNIFESP), São Paulo, Brazil.

[g] Schizophrenia Program (PROESQ), Department of Psychiatry, Universidade Federal de São Paulo (UNIFESP), São Paulo, Brazil

Corresponding author:

Prof. Dr. Michael Maes, M.D., Ph.D.

IMPACT Strategic Research Center

Barwon Health

Deakin University

Geelong, Vic

Australia

dr.michaelmaes@hotmail.com

https://scholar.google.co.th/citations?user=1wzMZ7UAAAAJ&hl=th&oi=ao

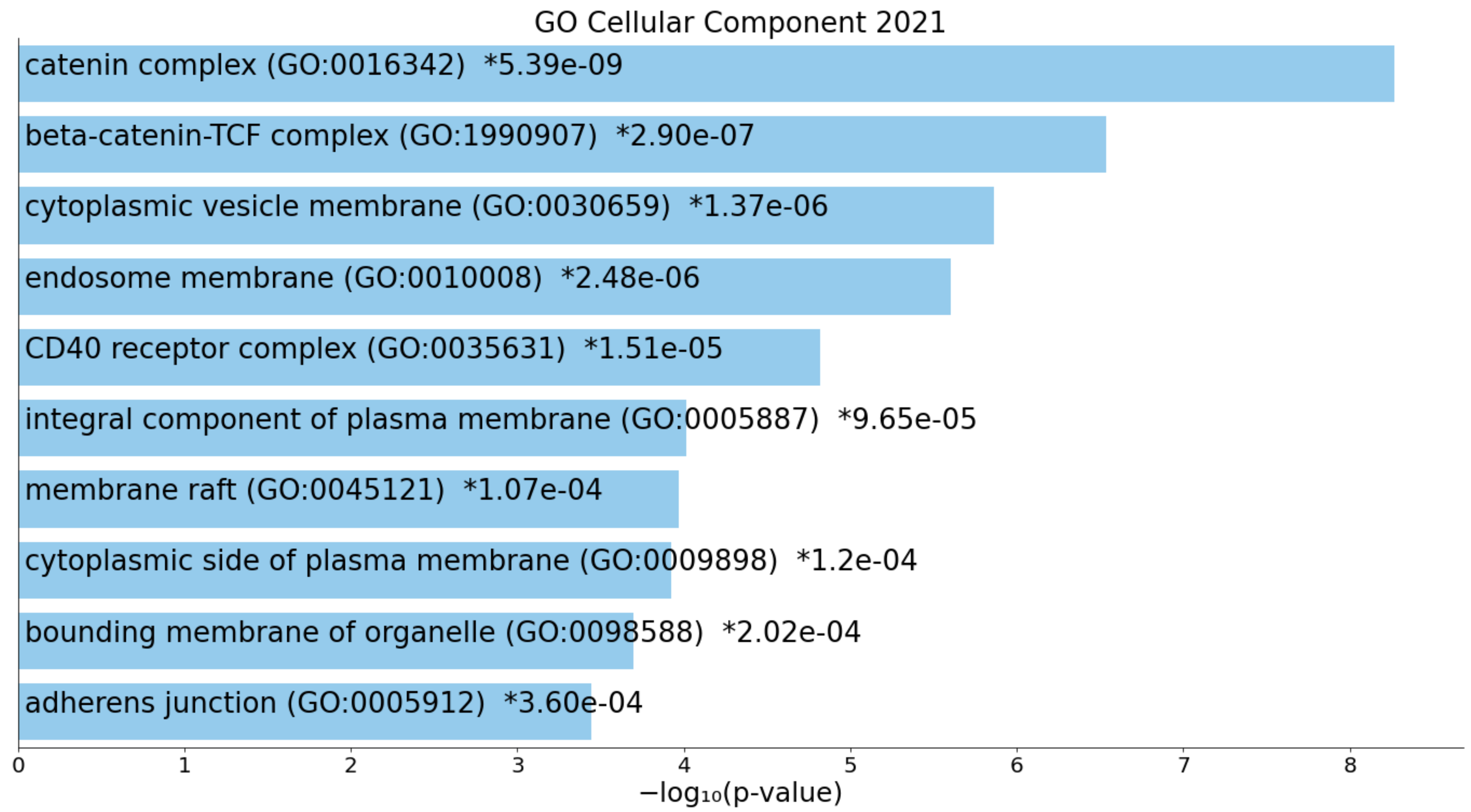

Figure S1. Heatmap (top-10) of enriched GO cellular component terms accumulated in the differently expressed proteins in first episode psychosis and schizophrenia.

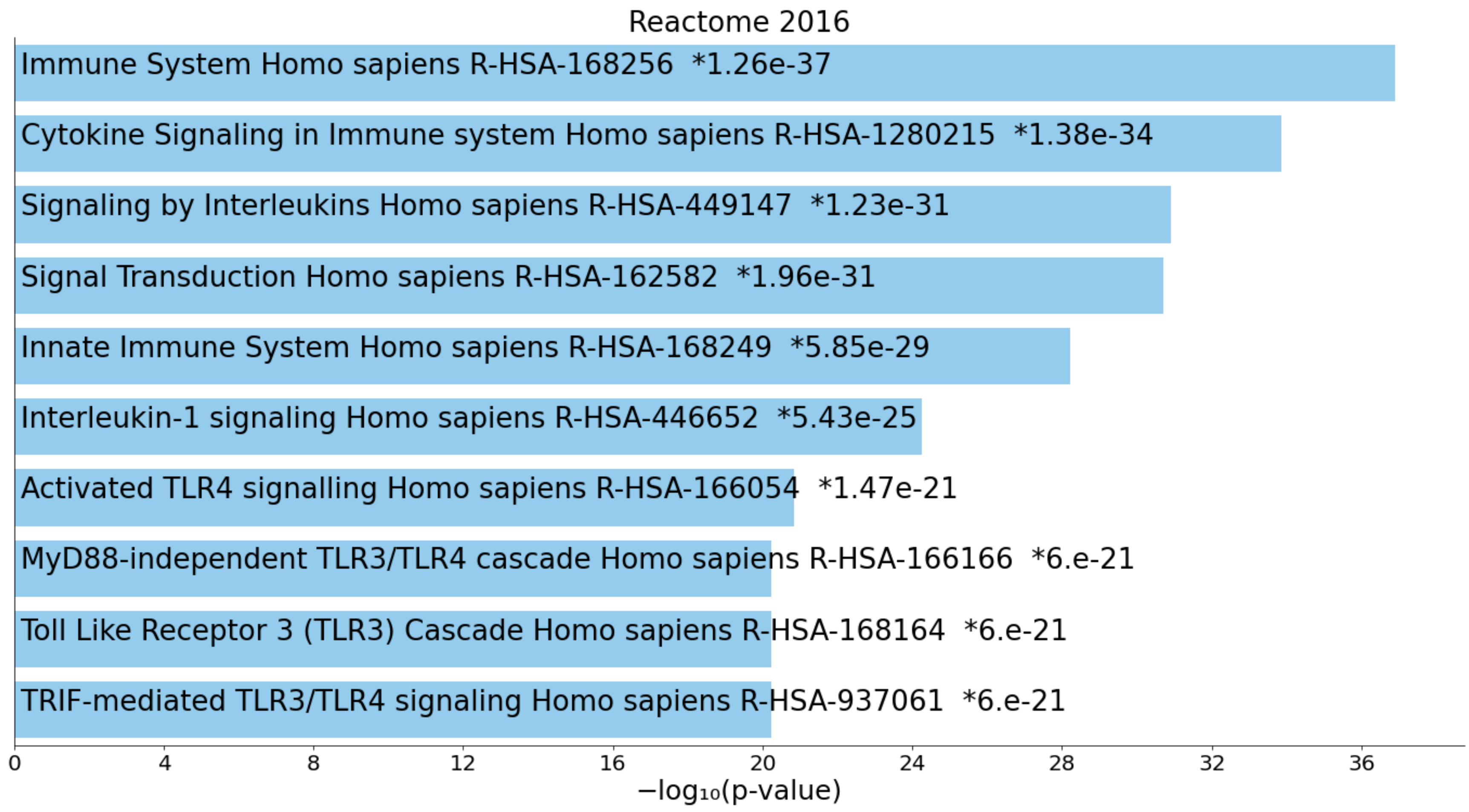

Figure S2. Heatmap (top-10) of enriched Reactome terms over-represented in the differently expressed proteins in first episode psychosis and schizophrenia.

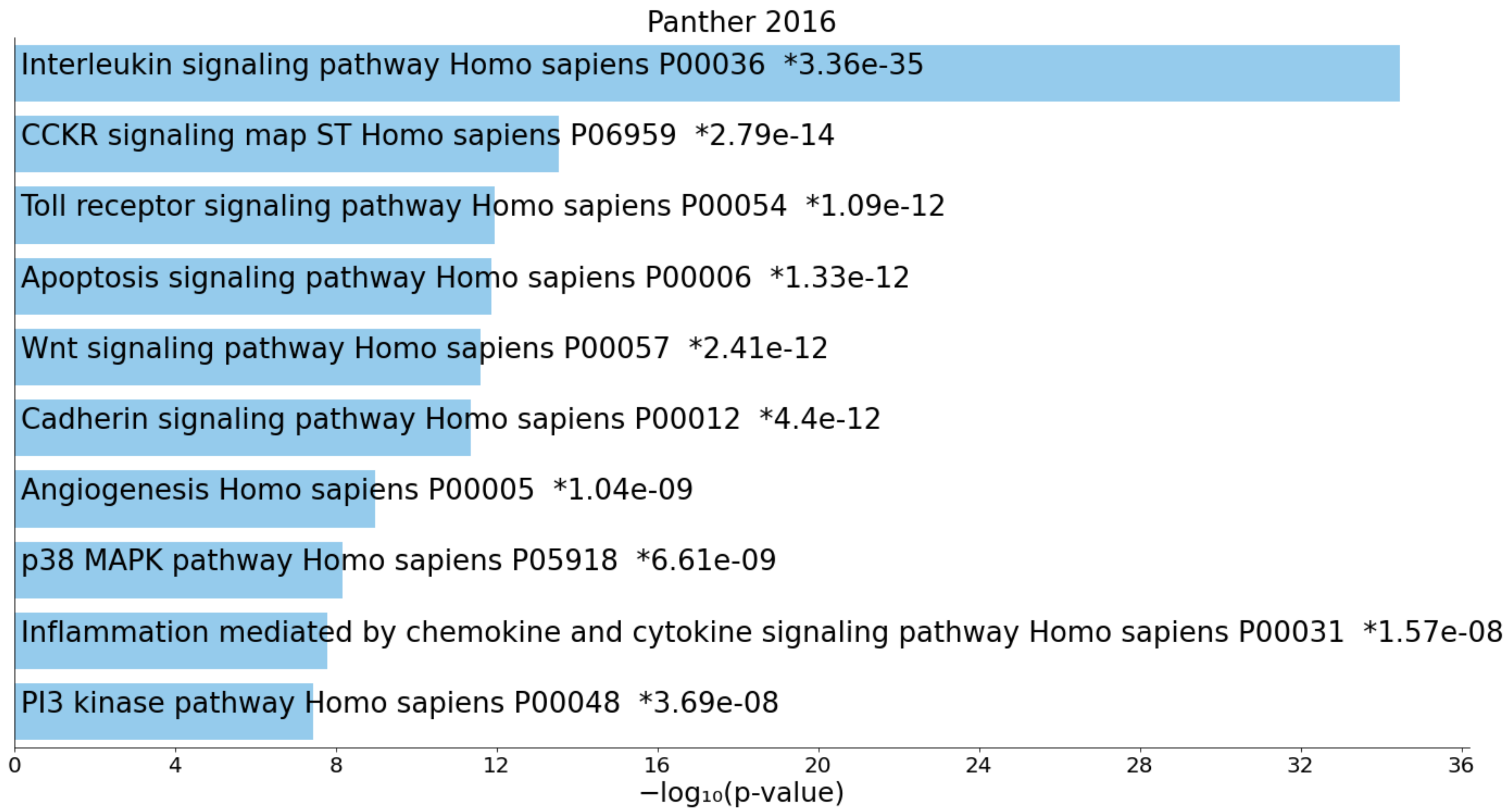

Figure S3. Heatmap (top-10) of enriched Panther Pathways that were over-represented in the differently expressed proteins in first episode psychosis and schizophrenia.

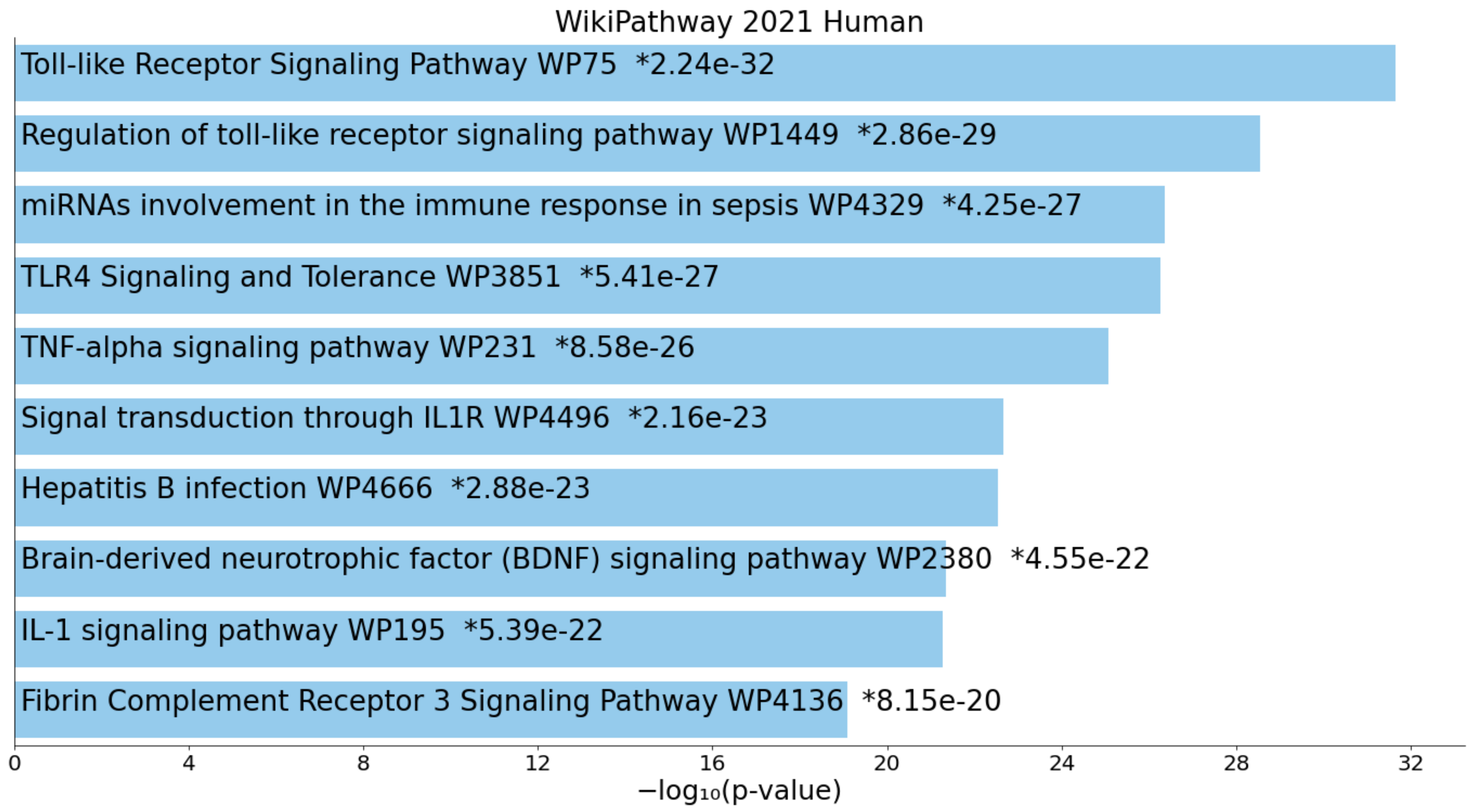

Figure S4. Heatmap (top-10) of enriched WikiPathways that were over-represented in the differently expressed proteins in first episode psychosis and schizophrenia.

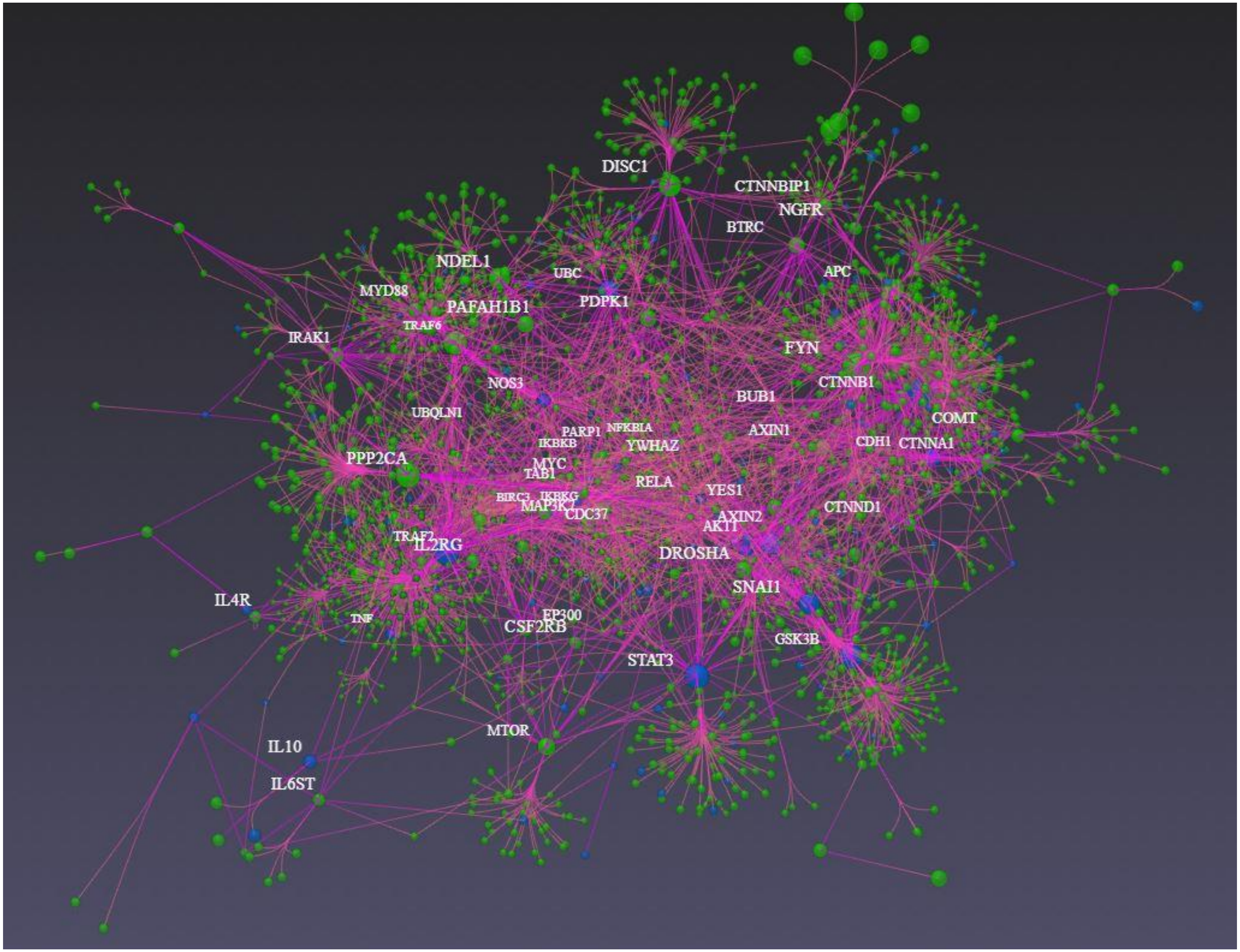
DISC1
CTNNBIP1
NGFR
BTRC
NDEL1
UBC
APC
MYD88
PAFAH1B1
PDPK1
TRAF6
IRAK1
FYN
NOS3
CTNNB1
BUB1
UBQLN1
COMT
PARP1
NFKBIA
AXIN1
IKBKB
YWHAZ
CDH1
CTNNA1
PPP2CA
MYC
TAB1
RELA
YES1
BIRC3
IKBKG
CTNND1
CDC37
AXIN2
AKT1
TRAF2
IL2RG
DROSHA
SNAI1
IL4R
EP300
TNF
CSF2RB
GSK3B
STAT3
MTOR
IL10
IL6ST

Figure S5. Protein-protein interaction network (built with OmicsNet and IntAct) of first episode psychosis and schizophrenia. The top-3 transcriptional factors are shown as blue spots (SP1, NFκB1, and RELA). Proteins in green and bundles in pink.

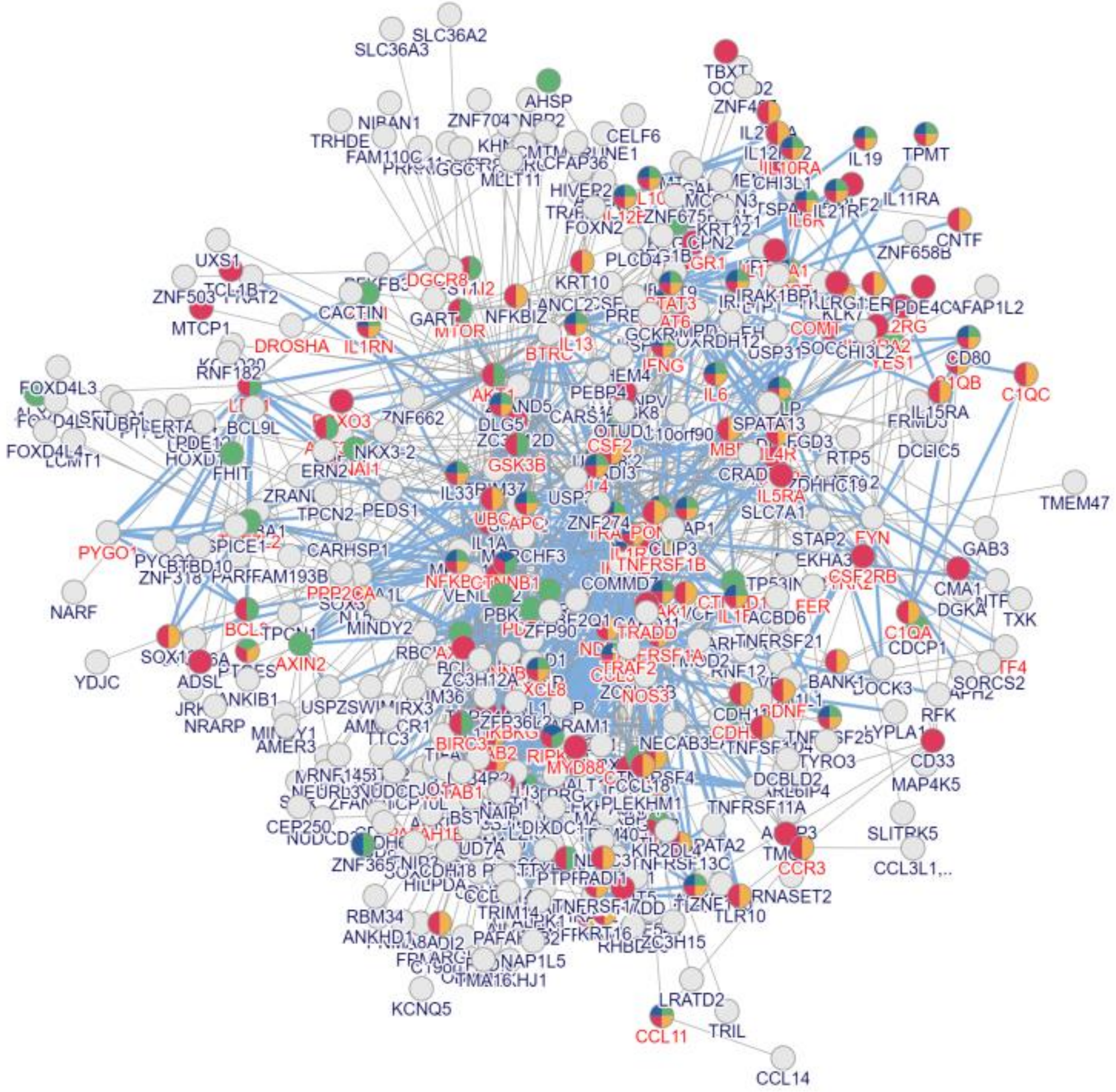

SLC36A2
SLC36A3
TBXT
AHSP
NIBAN1
TRHDE
CELF6
IL19
TPMT
IL10RA
IL11RA
CNTF
ZNF658B
FOXN2
KRT10
DGCR8
CACTIN
UXS1
ZNF503
MTCP1
DROSHA
IL1RN
MTOR
NFKBIZ
BTRC
IL13
STAT3
IFNG
IL6
COMT
CHI3L2
CD80
C1QB
C1QC
CAFAP1L2
IL15RA
FOXD4L3
FOXD4L4
BCL9L
ZNF662
GSK3B
NKX3-2
FHIT
ERN2
TPCN2
PEDS1
SPATA13
MBL
IL4R
RTP5
DCLIC5
TMEM47
CRADD
IL5RA
ZDHHC19
SLC7A1
STAP2
FYN
GAB3
CSF2RB
CMA1
DGKA
TXK
C1QA
CDCP1
SORCS2
PYGO1
NARF
ZNF318
CARHSP1
FAM193B
PPP2CA
NFKB
CTNNB1
COMMD7
TNFRSF1B
ZNF274
CLIP3
ZFP90
TRADD
TRAF2
NOS3
TNFRSF21
MINDY2
AXIN2
SOX13
ADSL
YDJC
JRK
NRARP
AMER3
TTC3
BIRC3
RIPK
MYD88
CXCL8
TAB1
TAB2
ZC3H12A
BANK1
BDNF
TNFSF4
TYRO3
CD33
MAP4K5
RFK
LYPLA1
DOCK3
DCBLD2
TNFRSF11A
PLEKHM1
CCL18
CCR3
SLITRK5
CCL3L1,..
CEP250
ZNF365
RBM34
ANKHD1
NAP1L5
TRIM14
PTPF
RNASET2
TLR10
ZC3H15
RHBDD
KCNQ5
LRATD2
TRIL
CCL11
CCL14

Figure S6. Protein-protein interaction network (built with inBio Discover) of differently expressed proteins in first episode psychosis and schizophrenia. Shown are 4 top annotations that are over-represented in the gene list, namely: red color: immune system disease (DOID:2914); blue color: inflammatory bowel disease (DOID:0050589); green color: intestinal disease (DOID:5295); yellow color: autoimmune disease (DOID:417)

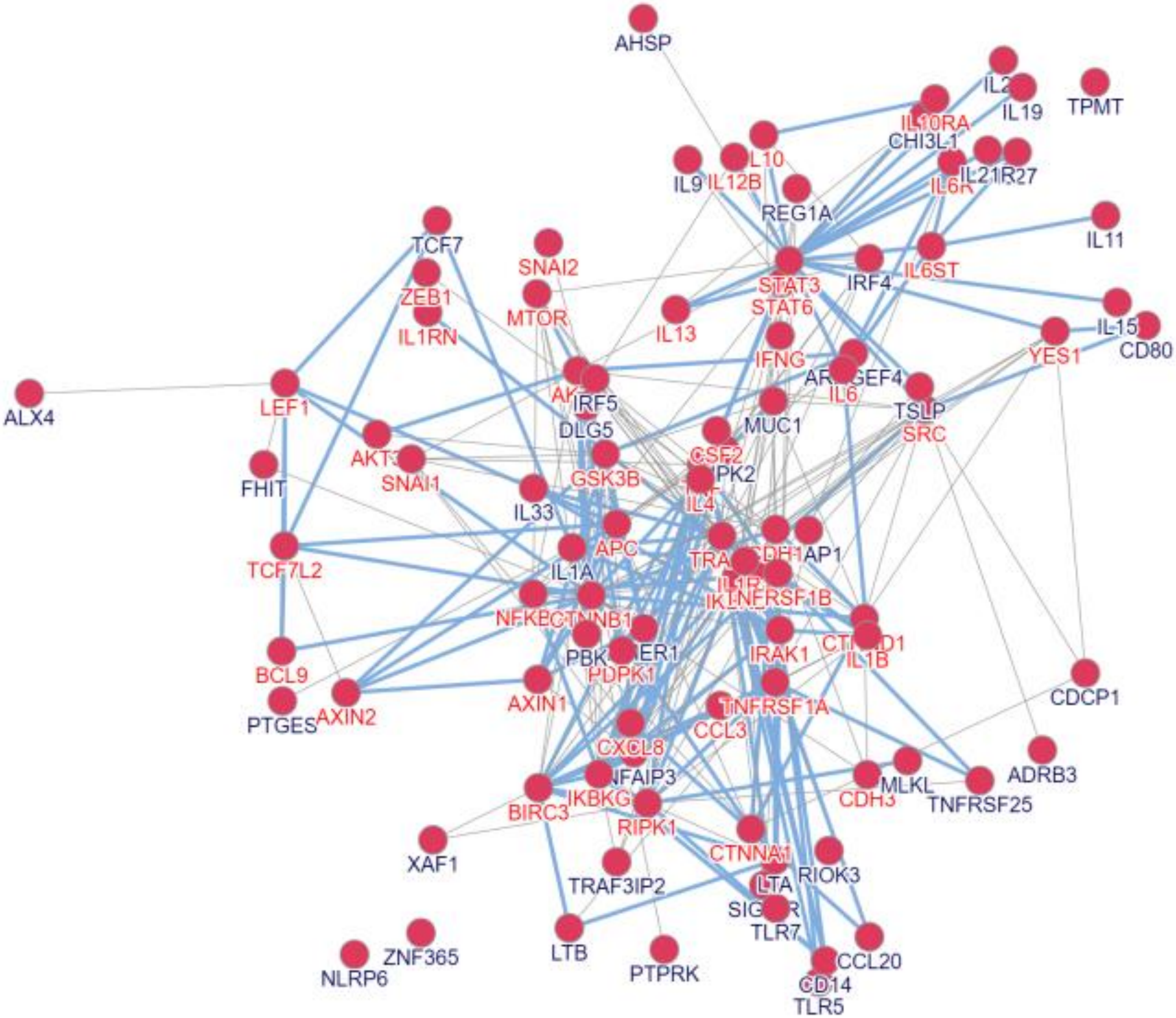

AHSP
IL19
TPMT
IL10RA
CHI3L1
IL10
IL9
IL12B
IL21R
IL27
IL6R
REG1A
IL11
TCF7
SNAI2
STAT3
IRF4
IL6ST
ZEB1
MTOR
STAT6
IL13
IL15
CD80
IL1RN
YES1
IFNG
ALX4
LEF1
IRF5
IL6
TSLP
MUC1
DLG5
SRC
AKT3
CSF2
SNAI1
FHIT
GSK3B
IL4
IL33
APC
AP1
TCF7L2
IL1A
NFRSF1B
CTNNB1
IRAK1
IL1B
BCL9
PDPK1
AXIN1
CDCP1
TNFRSF1A
PTGES
AXIN2
CCL3
CXCL8
ADRB3
MLKL
BIRC3
IKBKG
CDH3
TNFRSF25
RIPK1
CTNNA1
XAF1
RIOK3
TRAF3IP2
LTA
TLR7
LTB
CCL20
ZNF365
PTPRK
NLRP6
CD14
TLR5

Figure S7. Differently expressed proteins in the extended protein-protein interaction network that accumulated in intestinal disease (DOID:5295).

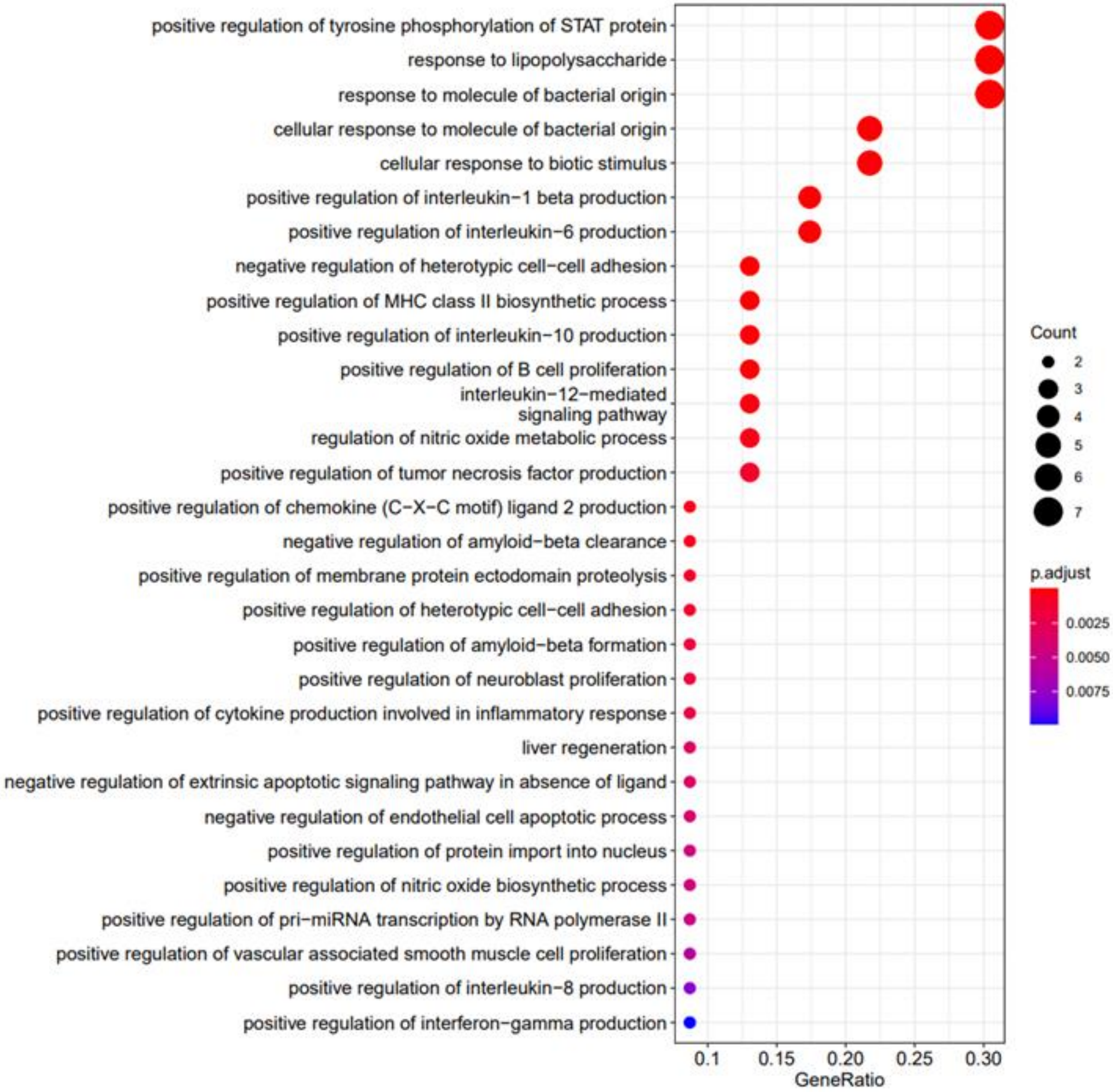

positive regulation of tyrosine phosphorylation of STAT protein
response to lipopolysaccharide
response to molecule of bacterial origin
cellular response to molecule of bacterial origin
cellular response to biotic stimulus
positive regulation of interleukin−1 beta production
positive regulation of interleukin−6 production
negative regulation of heterotypic cell−cell adhesion
positive regulation of MHC class II biosynthetic process
positive regulation of interleukin−10 production
positive regulation of B cell proliferation
interleukin−12−mediated signaling pathway
regulation of nitric oxide metabolic process
positive regulation of tumor necrosis factor production
positive regulation of chemokine (C−X−C motif) ligand 2 production
negative regulation of amyloid−beta clearance
positive regulation of membrane protein ectodomain proteolysis
positive regulation of heterotypic cell−cell adhesion
positive regulation of amyloid−beta formation
positive regulation of neuroblast proliferation
positive regulation of cytokine production involved in inflammatory response
liver regeneration
negative regulation of extrinsic apoptotic signaling pathway in absence of ligand
negative regulation of endothelial cell apoptotic process
positive regulation of protein import into nucleus
positive regulation of nitric oxide biosynthetic process
positive regulation of pri−miRNA transcription by RNA polymerase II
positive regulation of vascular associated smooth muscle cell proliferation
positive regulation of interleukin−8 production
positive regulation of interferon−gamma production
0.1
0.15
0.20
0.25
0.30
GeneRatio
Count
2
3
4
5
6
7
p.adjust
0.0025
0.0050
0.0075

Figure S8. GO functional enrichment analysis of all differently expressed proteins in first episode psychosis and schizophrenia using R package ClusterProfiler. Only annotated GO term leaves are shown in this figure. The x-axis shows the gene ratio and the y-axis the annotated GO biological terms. The size of the dots is proportional to the gene number; p-values of all GO terms are colored as indicated in the figure.
